# Strengthening the spiritual domain in palliative care through a listening consultation service by spiritual caregivers in Dutch PaTz-groups: an evaluation study

**DOI:** 10.1186/s12904-020-00595-0

**Published:** 2020-06-29

**Authors:** Hanna T. Klop, Ian Koper, Bart P. M. Schweitzer, Esli Jongen, Bregje D. Onwuteaka-Philipsen

**Affiliations:** 1 Department of Public and Occupational Health, Amsterdam UMC, Vrije Universiteit Amsterdam, Amsterdam Public Health research institute (APH), Amsterdam, the Netherlands; 2 Stichting PaTz, Alkmaar, the Netherlands; 3 Expertise Centre for Palliative Care, Amsterdam UMC location VUmc, Alkmaar, the Netherlands; 4 Spiritual caregiver, Netwerk Palliatieve Zorg Gooi & Vechtstreek, Gooi & Vechtstreek, the Netherlands

**Keywords:** Primary care, General practitioner, Spiritual care, Spiritual caregiver, Consultations, Nurses, Palliative care

## Abstract

**Background:**

Palliative care should be holistic, but spiritual issues are often overlooked. General practitioners and nurses working together in PaTz-groups (palliative home care groups) consider spiritual issues in palliative care to be relevant, but experience barriers in addressing spiritual issues and finding spiritual caregivers. This study evaluates the feasibility and perceived added value of a listening consultation service by spiritual caregivers in primary palliative care.

**Methods:**

From December 2018 until September 2019, we piloted a listening consultation service in which spiritual caregivers joined 3 PaTz-groups whose members referred patients or their relatives with spiritual care needs to them. Evaluation occurred through (i) monitoring of the implementation, (ii) in-depth interviews with patients (*n* = 5) and involved spiritual caregivers (*n =* 5), (iii) short group interviews in 3 PaTz-groups (17 GPs, 10 nurses and 3 palliative consultants), and (iv) questionnaires filled out by the GP after each referral, and by the spiritual caregiver after each consultation. Data was analysed thematically and descriptively.

**Results:**

Consultations mostly took place on appointment at the patients home instead of originally intended walk-in consultation hours. Consultations were most often with relatives (72%), followed by patients and relatives together (17%) and patients (11%). Relatives also had more consecutive consultations (mean 4.1 compared to 2.2 for patients). Consultations were on existential and relational issues, loss, grief and identity were main themes. Start-up of the referrals took more time and effort than expected. In time, several GPs of each PaTz-group referred patients to the spiritual caregiver. In general, consultations and joint PaTz-meetings were experienced as of added value. All patients and relatives as well as several GPs and nurses experienced more attention for and awareness of the spiritual domain. Patients and relatives particularly valued professional support of spiritual caregivers, as well as recognition of grief as an normal aspect of life.

**Conclusions:**

If sufficient effort is given to implementation, listening consultation services can be a good method for PaTz-groups to find and cooperate with spiritual caregivers, as well as for integrating spiritual care in primary palliative care. This may strengthen care in the spiritual domain, especially for relatives who are mourning.

## Background

Spiritual care is an important part of holistic palliative care [1]. In the Dutch guideline on ‘Spirituality and meaning in the last phase of life’, spirituality is defined as “the dynamic dimension of human life that relates to the way persons experience, express and/or seek meaning, purpose and transcendence, and the way they connect to the moment, to self, to others, to nature, to the significant and/or the sacred” [2]. Spiritual care covers a multidimensional field of [1] existential issues, e.g. identity, meaning, suffering and death [2]; considerations and values, e.g. important issues and values for oneself, and [3] religious considerations and sources, e.g. faith, beliefs and practices [2, 3]. Receiving spiritual care as part of holistic spiritual care is associated with positive effects on patients’ quality of life and well-being. Patients and their relatives are open for discussing spiritual issues with their healthcare provider and appreciate these conversations. On the other side, a lack of spiritual care by healthcare professionals is associated with, amongst others, a poor quality of life [4–13].

Insufficient attention for spiritual issues has been identified as one of the barriers in providing holistic palliative care in the Netherlands [14, 15]. Palliative care is not a medical specialty in the Netherlands and it is preferably provided in the primary care setting, where it primarily falls under the responsibility of general practitioners (GPs) in close collaboration with district nurses. In respect of palliative care, Dutch GPs are encouraged to work together with district nurses and other healthcare providers in local PaTz-groups (palliative home care groups) [16]. PaTz-groups meet at least six times per year under supervision of a palliative care consultant [17]. The goal of these meetings is to identify patients facing a life-threatening illness, e.g. by using the surprise question, and to discuss current and future care needs of these patients, and to arrange and plan care accordingly.

Although spiritual issues are considered relevant by Dutch GPs [18], they often struggle to provide adequate spiritual care, due to lack of time or attention for spiritual issues, or insufficient expertise and training [19]. When a GP finds him- or herself unable to provide adequate spiritual care, for example in a case with complex spiritual needs or when crisis intervention is needed, they can theoretically refer their patient to a professional spiritual caregiver, a healthcare provider with specific expertise in the assessment of spiritual needs and the delivery of spiritual care [2, 20]. In reality, only half of the Dutch GPs occasionally involve a spiritual caregiver. When asked what reasons they have to not refer patients to spiritual caregivers, GPs mention that it is ‘not needed’, ‘not my job’, or that they ‘do not know where to find them’ [21]. In addition, they experience barriers in finding and engaging spiritual caregivers [18], and cooperation between spiritual caregivers and other healthcare providers has been reported to be poor [19, 22]. Thus, in practice, although providing spiritual care as part of holistic palliative care in cooperation with spiritual caregivers is considered to be very relevant and essential, it is often neglected and problematic, a phenomenon which has also been reported in international literature [23–26].

There is anecdotal evidence with regard to involving spiritual caregivers in palliative care in order to strengthen the spiritual domain of palliative care. In Scotland, a Chaplain Community service aimed at seriously ill patients, known as listening consultations, was perceived by patients, GPs and spiritual caregivers to be very beneficial. Activities of spiritual caregivers included therapeutic listening; being present; recognition of fear, loss and sadness; building trustful relationships in which difficult topics could be discussed; and helping patients to find hope, resilience or inner strength in times of illness, loss and death [27]. Knowing each other and each other’s activities well proved to be pivotal for the cooperation between spiritual caregivers and other healthcare providers [27].

In the Netherlands, the above-mentioned PaTz-groups may provide an opportunity for fruitful cooperation between healthcare providers and spiritual caregivers, potentially resulting in improved spiritual care in the primary care setting. With the Chaplain Community project serving as an example, the PaTz-foundation launched a pilot in which a listening consultation service by spiritual caregivers was connected to GPs and nurses in PaTz-groups in an effort to strengthen the spiritual domain in Dutch primary palliative care.

In this explorative study, we aimed to evaluate and describe the listening consultation service with regard to its feasibility and perceived added value for healthcare providers and patients. Our research questions were:

1. How did the process of implementation of a listening consultation service within PaTz-groups go, and what are barriers and facilitators for implementation?

2. What is the perceived added value of a listening consultation service for healthcare providers and for patients?

## Methods

### Design

**Fig. 1:**
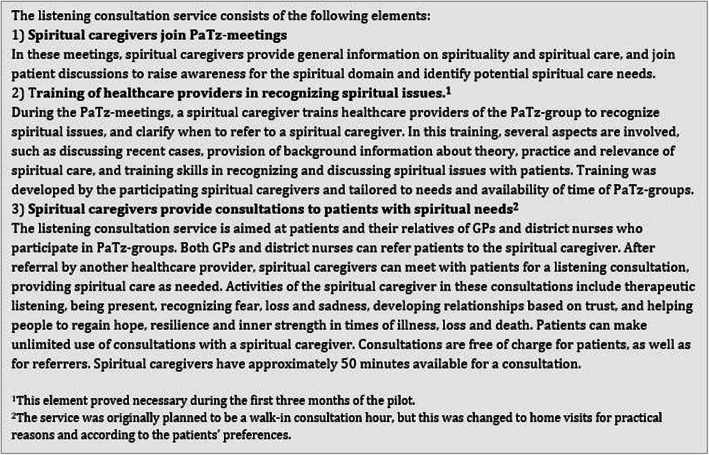
Overview of the intervention: Listening consultation services

For a period of ten months, the listening consultation services ran in three PaTz-groups. Qualitative data for the evaluation of the listening consultation service was collected through questionnaires as well as individual and group interviews, supplemented with quantitative data on personal and consultation characteristics. A description of the intervention is shown in Fig. 1. The consolidated criteria guidelines for reporting qualitative studies (COREQ) were followed for reporting on qualitative data [17].

### Recruitment for the intervention

PaTz-groups were recruited via the PaTz-foundation and via the Palliative Care Consortium in Noord-Holland and Flevoland. Chairmen of four interested PaTz-groups were provided with information during a meeting with the researcher and after consenting to participate in the pilot, the researcher visited a meeting of each PaTz-group in which the spiritual caregiver was introduced. In these meetings, the researcher provided all PaTz-group members with flyers which could be handed out to patients. Finally, three interested PaTz-groups participated in the pilot.

Spiritual caregivers were included in collaboration with the ‘Center for Life Questions’ (in Dutch: Centrum voor Levensvragen), who selected interested spiritual caregivers based on their availability and their fit to the patient population of involved general practices. In total, seven spiritual caregivers were recruited (two per PaTz-group, with one backup and one who was recruited after drop-out of another spiritual caregiver). Four of them had a humanistic denomination, one an Islamic denomination, one Christian and one Buddhist. All spiritual caregivers were affiliated with the Dutch Association of Spiritual Caregivers (VGVZ), which is the national professional association of spiritual caregivers with approximately 1000 members who are currently employed in a care facility or in primary care. This association uses a professional standard for providing spiritual care. All spiritual caregivers were registered in the Quality Register of Spiritual Caregivers (SKVG), an association that manages the professional register of spiritual carers. The purpose of this register is to guarantee the professional level of the profession and to promote social recognition. All spiritual caregivers had to be available during planned PaTz-meetings.

During the study, patients and relatives with spiritual care needs were asked by the participating GPs whether they were interested in receiving the listening consultation service, or informed by a flyer in the waiting room. If so, they were provided with information, and were asked for permission to be approached by the spiritual caregiver attached to the GP’s practice. The spiritual caregiver then contacted the patient for a first introduction and schedule a meeting. Patients and relatives who received the listening consultation services could also involve their relatives. Consultation with patients or relatives were free of charge. For time spend on consultations and attendance at PaTz-groups, spiritual caregivers were reimbursed from the project budget.

### Data collection

Data was collected from December 2018 until September 2019. Several qualitative and quantitative data collection methods were used to collect data on several aspects of the implementation process, the intervention and the perceived added value. Questionnaires with structured and open questions on characteristics and content of the consultation were filled out by spiritual caregivers after each consultation, which resulted in an overview of characteristics of users of consultations, and content of consultations. Additionally, at the end of the pilot study, questionnaires with structured and open questions on experiences with and perceived added value of spiritual care were filled out by referrers for all patients and relatives who used the listening consultation service. For the qualitative part, all participants were recruited by opportunity sampling and informed by an information letter. Semi-structured in-depth interviews were held with spiritual caregivers (*n* = 5) at the end of the pilot study or when they were not longer involved in the pilot study. In addition, semi-structured in-depth interviews were held with patients and relatives (*n =* 5). They were recruited by the spiritual caregiver, who provided an information letter and asked if the patient was willing to participate in an interview on experiences with the consultation service. If the patient or relative was interested, the spiritual caregiver provided contact details to the researcher, who contacted and informed the patient by phone. With their consent, an appointment for the interview was made. Finally, short group interviews were held with PaTz-groups at the end of the pilot study (*n =* 3, with 17 GPs and 10 nurses). All interviews were performed by one female researcher in palliative care (HK) who was trained in qualitative research, and were conducted at the participants’ location of choice. Duration of interviews was between 20 and 60 min for patients, between 40 and 70 min for spiritual caregivers and between 20 and 45 min for PaTz-groups. No participants refused to participate. All interviews were guided by semi-structured topic lists (Additional file 1), and audio recorded and transcribed verbatim. A weekly diary was used to monitor the implementation process in this pilot. The characteristics of all participants in the interviews are presented in Table 1.

**Table 1:** Characteristics of participants involved in data-collection

Type of participant(s)	Type of data-collection method	Sex	Age range (years)	^**c**^ / denomination^**b**^
Relative of deceased family member, patient of GP	Individual interview	F	50–55	None
Relative of deceased family member, patient of GP	Individual interview	F	55–60	None
Relative of deceased family member, patient of GP	Individual interview	F	20–25	None
Relative of (deceased)^c^ family member, patient of GP	Individual interview	M	75–80	Christian, other
Relative of deceased family member, patient of GP	Individual interview	F	60–65	Buddhist
Spiritual caregiver	Individual interview	F	60–65	Humanistic
Spiritual caregiver	Individual interview	M	35–40	Islamic
Spiritual caregiver	Individual interview	F	60–65	Humanistic
Spiritual caregiver	Individual interview	F	55–60	Buddhist
Spiritual caregiver	Individual interview	F	30–35	Humanistic
7 GPs, 5 district nurses, 1 palliative care consultant	Group interview	4 M9F	N/A	N/A
5 GPs, 5 district nurses, 1 nurse specialized in palliative care, palliative care consultant	Group interview	2 M10F	N/A	N/A
3 GPs, 4 district nurses, palliative care consultant	Group interview	8F	N/A	N/A

^a^In case of patients / relatives who had one or more consultations with the spiritual caregivers

^b^In case of spiritual caregivers

^c^ Family member deceased during the pilot period

### Data analysis

Various methods of analysis were used to involve several perspectives in the analysis. After answers to open questions were categorized by one researcher (IK) and checked by a second (HK), descriptive analyses took place for quantitative data using SPSS 26.0. Qualitative data of the semi-structured interviews with spiritual caregivers, patients and relatives, and health care professionals in PaTz-groups, were analysed following the principles of thematic analysis [28]. After rereading transcripts, one researcher (HK) derived codes inductively from the data using Atlas.ti 8. The codes were checked by a second researcher (IK). Interpretation of the codes and themes was discussed regularly between HK, IK and BO to ensure peer debriefing and to function as a mechanism against interpretation of one single researcher. Negative case analysis was also included to ensure that results were based on both positive and negative attitudes towards aspects of the intervention or the intervention as a whole. Finally, codes were grouped into themes and all themes were discussed in the research team, until no new themes occurred (HK, IK, BO).

### Ethics

Patients, spiritual caregivers and healthcare providers who participated in the interviews gave written informed consent prior to the interview. Patients received a gift voucher for their participation. To ensure anonymity of participants, any personal identifying information was removed from the transcripts. Access to the data was limited to three researchers (HK, IK, BO).

## Results

First, characteristics of the implementation process of listening consultation services including involvement of a spiritual caregiver to PaTz-groups and experienced facilitators and barriers are described. Second, the perceived added value and experiences regarding the listening consultation services and involvement of spiritual caregivers to PaTz-groups, are provided.

### Implementation of the intervention

#### Process of involvement of spiritual caregivers in PaTz-groups

Spiritual caregivers attended eleven of fourteen PaTz-meetings during the pilot period. They were involved in patient discussions, asked questions concerning spiritual issues to the present GPs and nurses, and answered their questions. The time investment and effort required from spiritual caregivers turned out to be more than expected, due to the time-intensive start-up of referrals. The combination of collaboration between GPs, nurses and spiritual caregivers in PaTz-groups, and possibilities of referring to a spiritual caregivers hardly led to referrals. For GPs and nurses, recognition of spiritual issues appeared to be a barrier in this. Therefore, in all PaTz-groups a spiritual caregiver provided at least one training in recognizing and discussing spiritual issues in patients, as well as referring to spiritual caregivers. Further, activities such as (preparation for) training and meetings with other spiritual caregivers and the researcher needed time and effort in the beginning phase of the pilot.

#### Process of referrals

The consultation process started slowly, but consultations became more frequent during the pilot period. Consultations had been offered to both patients and relatives, but, we found that mainly relatives made use of the possibility of consultations. Referral took place not only by GPs, but also by relatives who used or had used consultations. After the training given by a spiritual caregiver, the numbers of referrals increased over time.

#### Characteristics of consultations

Spiritual caregivers held 46 consultations with 19 individuals (patients with a life-threatening illness and their relatives) with an mean age of 73. The majority was female (15/19), had no specific religious beliefs (8/19) or a Christian belief (4/19), were of Dutch descent (18/19) and were referred by their GP (13/19) or a family member (6/19). Nurses did not refer at all. They did not indicate a clear reason for this, except that they thought the patient had sufficient resources for spiritual care. The listening consultation service was used mainly by relatives (13/19) of terminal or deceased patients. Although the initial idea was to organise walk-in consultation hours in general practice, this turned out not to be feasible; home visits were the preferred alternative. Mainly one-to-one consultations were held (33/46), but group consultations with several relatives (7/46) or relatives and patients (6/46) were also held in varying compositions. Relatives used more consecutive consultations (M = 3.6) than patients (M = 1.8). Most care requests contained existential (32/46) or relational (24/46) components. The most often discussed topics were loss, grief and identity. Table 2 provides an overview of characteristics of participants who had consultations with the spiritual caregivers.

**Table 2:** Characteristics of participants who had consultations with spiritual caregivers

	n/N	Mean (range)
Total unique users		
Patients	6/19	
Relatives	13/19	
Sex		
Female	15/19	
Male	4/19	
Conviction		
None	8/19	
Christian	4/19	
Other	3/19	
Unknown	4/19	
Nationality		
Dutch	18/19	
Surinamese	1/19	
Referred by		
GP	13/19	
Relative (already involved)	6/19	
Total unique consultations	46	
First consultation	14/46	
Follow-up consultation	32/46	
One-to-one consultations	33/46	
Patients at the end of life	5/33	
Relatives	28/33	
Group consultations	13/46	
Patient and relative	6/13	
Multiple relatives	7/13	
Consultations per user		3.1 (1–10)
Per patient		1.8 (1–3)
Per relative		3.6 (1–10)
Lengths of consultations (minutes)		69 (15–150)
One-to-one consultations (minutes)		62 (15–100)
Group consults (minutes)		89 (45–150)
Care request^a^		
Existential	32/46	
Relational	24/46	
Psychological	12/46	
Religious	3/46	
Discussed topics^b^		
Grief	33/46	
Loss	30/46	
Identity	24/46	
Death / passing away	22/46	
Support	21/46	
Meaning	19/46	
Fear	18/46	
Finding strength	18/46	
Hope	11/46	
Other / diverse	33/46	

^a^ Participants could have more than one care request

^b^ Spiritual caregivers could report more than one discussed topic

**Fig. 2:**
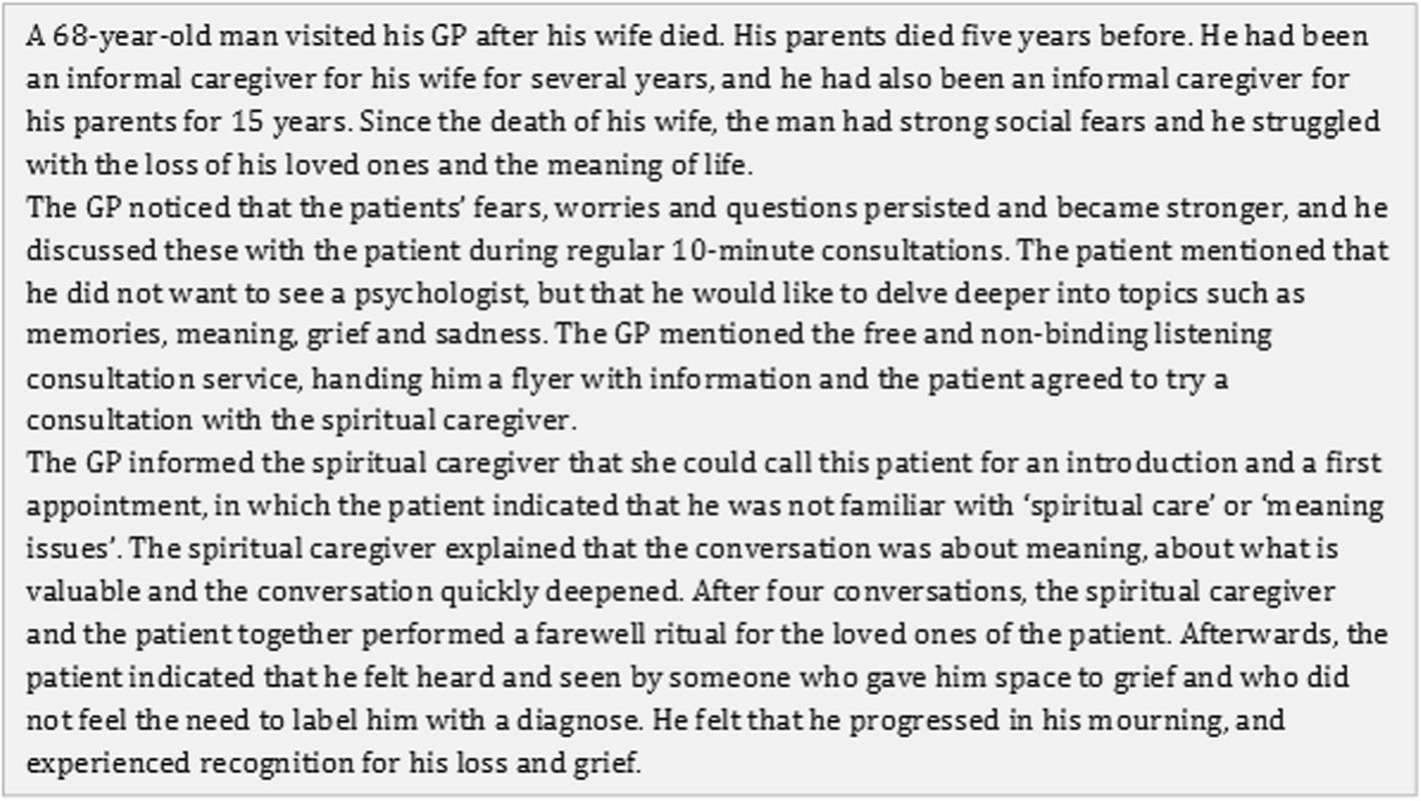
Practical example of a consultation of a spiritual caregiver by a patient

A practical example of a consultation of spiritual caregivers by a patient is provided in Fig. 2.

### Facilitators and barriers for the listening consultation service

#### Facilitators for implementing spiritual care into palliative care in primary care

First, frequent contact between spiritual caregivers and healthcare providers, in terms of spiritual caregivers attending PaTz-groups regularly and providing feedback about consultations to the referrer, proved to be a facilitator for implementation. This frequent contact resulted in health care providers more often thinking of spiritual issues and also referring patients more easily. Second, customization and flexibility in setting up listening consultation services and involvement of spiritual caregivers proved to be encouraging, e.g. consultations by home visits or a focus on relatives as a target group. This resulted in a service that was feasible and useful for those involved. Third, a project manager proved to be valuable when integration of spiritual care into already existing PaTz-groups took more time and effort than expected. Fourth, freedom of spiritual caregivers, e.g. consultations on appointment instead of a walk-in consultation hour or an unlimited number of consultations, was found to be a facilitator, as were the enthusiastic PaTz chairmen. Lastly, training given by spiritual caregivers on recognizing spiritual issues and discussing them with patients, was found to facilitate the integration of spiritual issues in primary palliative care.

#### Barriers for implementing listening consultation services

Some factors impeded the implementation and functioning of the listening consultation service. Spiritual caregivers mentioned that they struggled to get into contact with PaTz-groups and that the PaTz-groups did not meet as regularly as expected. The other healthcare providers mentioned the limited availability and occasional last-minute cancellations of spiritual caregivers as barriers for cooperation. Also, the terms ‘spirituality’ and ‘spiritual care’ turned out to be barriers, as these were often associated with religion or were difficult to concretize. Furthermore, some GPs had privacy concerns when referring a patient to a spiritual caregiver. Finally, the relatively short pilot period turned out to be a barrier, as spiritual caregivers and healthcare professionals needed more time to get to know and find each other, and, addressing and recognizing spiritual issues by GPs increased over time.

### Perceived added value of the listening consultation services

Experiences of healthcare professionals, patients and patients’ relatives and spiritual caregivers, are illustrated by quotes in Table 3.

**Table 3:** Quotes on the perceived added value of the listening consultation service

Theme	Quote
For HCP’s	*“R1: The fact that she [spiritual caregiver] is involved in PaTz-groups, means that you think of it as well, you see someone, and then you think of consultations, and of a different perspective, or of some extra possibilities. R2: R2: Because here [PaTz-group meeting] you sometimes can get stuck in the medical issues. Or when you have issues with providing care to a patient or whatever, and then you can say: maybe it’s an idea that …*” *(GPs in PaTz-group)*
For patients /relatives	*“Well, it just seems a bit like a soft way to talk about loss, without having a label or something, I experienced that as very pleasant. Because sometimes I have discussed these issues with my friends or sometimes family when I felt sad at certain moments. Then they say that you should just take a pill. Sometimes I have received that advice. Or that I had to engage a psychologists. But I don’t think I want that at all. That’s not the point at all. Then you feel somewhat misunderstood. And then I’d rather talk to an expert about it.” (Patients’ relative, 50y)*
For spiritual caregivers	*“Maintaining the part of communication with each other, that has had quite a lot of attention in such a start-up phase. (…*) *And then, I just think, that is worth the investment you know, if you can find each other well at the moments that matter. And if patients sometimes appreciate it if you give something back to the GP, yes, then you just work together on good, holistic patient care. So sometimes the investment is that it costs you extra time, but I think it is definitely worth it over time..” (Spiritual caregiver involved in pilot)*

#### Experiences of healthcare professionals participating in PaTz-groups

Enthusiasm for and perceived value of the listening consultation services varied per healthcare professional. Most GPs and district nurses who participated in the PaTz-groups felt the listening consultation services’ added value. Firstly, the additional expertise of spiritual caregiver provided them with a broader perspective on the patient or relative(s) which was often focused on (psycho) social and spiritual wellbeing of the patient. Secondly, healthcare professionals felt that the listening consultation services facilitated identification and discussion of spiritual issues with the patient, although addressing spiritual issues in daily practice remained difficult. Also, healthcare professionals mentioned that spiritual caregivers sometimes spoke a “different language”. A small amount of GPs and district nurses did not experience added value of the listening consultation services, most often due to available alternatives, such as a centre for relief and support for people with cancer, a nurse specialist in mental health (POH-GGZ), or because of their own perceived capacities in the field of spiritual care.

#### Experiences of patients and relatives using the listening consultation services

Interviewed relatives who had one or more consultations with spiritual caregivers, perceived the conversations as very valuable, especially in the recognition of normal feelings in times of loss and the recognition of grief as a normal aspect of life. Also, they experienced added value when they were mourning, and they appreciated this low-threshold and free initiative of listening consultation services. In particular, the role of the spiritual caregiver as a “humane person” who provides professional support, was well-appreciated. In addition, according to PaTz-group members, their patients perceived the consultations as valuable in a similar way as relatives did.

#### Experiences of spiritual caregivers who were involved in consultations and PaTz-groups

Spiritual caregivers felt that the listening consultation service added to holistic palliative care and the possibility to integrate spiritual care as a professional discipline into palliative care. They felt that the nature of care requests and discussed themes with patients fitted their mission as a spiritual caregiver: using listening as an essential and important instrument, providing meaning to illness and life stories, and connecting relatives and patients to God, religion or the ultimate, and providing support and rituals. At the same time, spiritual caregivers experienced that they needed time to become familiar with the PaTz-group members and their professions, and that integrating spiritual care into palliative care took more time and effort than expected. As a positive side effect of this intervention, some spiritual caregivers mentioned increased knowledge of possibilities for spiritual care among healthcare professionals, as well as increased reach of patients in palliative care.

## Discussion

### Summary of results

This study evaluated and described a pilot of a listening consultation service by spiritual caregivers in PaTz-groups. It showed that, although time-intensive and difficult at start, the intervention could be feasible and has added value. Consultations were on existential and relational issues, mainly on loss, grief and identity were main themes. After a period of gaining momentum, the listening consultation services resulted in more attention for spiritual issues of patients and relatives in particular, who both highly appreciated this. Healthcare providers in PaTz-groups, particularly GPs, were more aware of (addressing) spiritual issues that could be relevant for their patients. They also experienced added value in the complementary expertise of spiritual caregivers. Still, the enthusiasm among GPs varied and nurses did not refer patients to spiritual caregivers at all. Involving spiritual caregivers in PaTz-groups seemed to be a good method to improve spiritual care in the primary care setting and cooperation between healthcare professionals and spiritual caregivers.

### Scarce evidence on integrating spiritual care in palliative care

Previous research has shown that healthcare professionals consider spiritual issues in palliative care to be relevant [29, 30], and that attention for spiritual issues positively affects the patients’ relationship with their care provider, reduces discomfort, and increases quality of life [29, 31]. This is, to our knowledge, the first intervention in which spiritual caregiver consultations of patients with a life-threatening illness and relatives, are combined with training of healthcare professionals in spiritual issues as well as enhanced collaboration between primary healthcare providers and spiritual caregivers. This study provides useful insights into the integration of spiritual care in primary palliative care, and into the perceived added value of listening consultation services from the perspectives of patients and relatives, and all healthcare professionals that are involved.

### Training as core element of integrating spiritual care into palliative care

Several European studies have shown that spiritual caregivers can play an important role in the training of other healthcare providers to discuss spiritual issues in palliative care [29]. Training turned out to be an essential part of this intervention, especially in the ability of healthcare providers to recognize and address spiritual issues in patients. Despite the relatively small set-up of this pilot study, our results indicate that training combined with close collaboration and regular meetings between GPs, nurses and spiritual caregivers gradually resulted in more awareness of and skills in spiritual care. Our results with regard to training and education are in line with other studies, that showed some positive effects of training hospice staff and hospice volunteers [32–34].

### Comparison to the Scottish chaplain community listening services

Similar to the Scottish Chaplain Community Listening service [27], our pilot study showed unfamiliarity of healthcare professionals with spiritual issues. Healthcare providers found it difficult to recognize and address spiritual issues of patients, and often associated spiritual issues with religious care, like in the Scottish example. Also similar to the Scottish example was that this intervention seemed a good alternative for psychotherapy or counselling when patients experienced mourning and feelings of sadness due to (nearing) loss of a loved one. Both patients in the Scottish and Dutch situation appreciated this type of spiritual care particularly because of the non-judgemental and non-stigmatizing approach. This could be helpful for patients who do not want to be referred to other specialists or care providers. Lastly, similar to the Scottish situation, Dutch patients valued “safe space” by visits and availability of time of spiritual caregivers, as well as an unlimited number of consultations and no obligations, as positive. In addition, our study showed the benefits of a combined approach that consists of a spiritual caregiver using existing infrastructure in palliative care such as PaTz, and consultations with patients by the same spiritual caregiver. This combined approach enhanced close collaboration between healthcare providers and spiritual caregivers as well as competency and skills of especially GPs in providing holistic palliative care.

### Financial considerations

In this pilot, the time spiritual caregivers spent attending PaTz-meetings, training healthcare providers and in consultations with patients and relatives was funded from the projects budget, removing a potential hurdle for healthcare providers and patients to make use of the listening consultation service. Without funding, the accessibility of these services may be limited as people may not be willing or able to pay for these consultations. In 2018, a governmental budget for consultations with spiritual caregivers, training and activities to increase awareness of the (possibilities of) spiritual caregivers was introduced for a period of 2 years [20]. Since 2019, this budget has been managed by local networks of palliative care [35]. As a result, financing this care does not have to be a barrier to the deployment of spiritual care in the Netherlands. In other countries, if there is no financial arrangement for spiritual care, this could be a barrier.

### Strengths and limitations

A strength of our study is that we were able to adapt the intervention to the needs of the participants, adding training for healthcare providers and substituting the walk-in consultation hours for home visits. By these changes, this study offers insight into facilitators and barriers to the provision of holistic palliative care, resulting in a list of practical recommendations before starting an intervention focused on the integration of spiritual care in palliative care (Additional file 2).

No baseline data was collected, which could be seen as a limitation as stating “added value” could also be of other reasons. However, since the aim of this study was to describe the process and perceived added value in a mainly qualitative and descriptive way, we explored a need and feasibility for this intervention and how to design this intervention. We believe that baseline data collection would be a good second step in the further development of this intervention. Also, the small scale of the pilot impacts the generalizability of the results. Although the listening consultation services seemed beneficial in the three participating PaTz-groups, this may be different in other PaTz-groups that may be less receptive to spiritual care. In addition, due to the small-scaled piloted intervention and explorative nature of the data, we did not collect large-scaled data on which we could establish significant relationships and influences, e.g. on perspectives on spirituality versus age and gender. More research is needed to understand large-scale provision of spiritual care in primary palliative care, also in a wider context such as other countries. Further, the district nurses in this study did not refer to spiritual caregivers, but we found no clear explanation for this. It may be related to their perspective on spirituality, or on their own resources on providing spiritual care or referring to spiritual caregivers in their own network. However, we did not conduct additional interviews due to lack of budget and time. We recommend further investigation of the role, perspectives and resources of (district) nurses regarding spirituality in palliative care in future research. Also, this study used mainly qualitative or descriptive quantitative methods in order to monitor implementation and to evaluate perceived added value. A more rigid, large scale method such as an RCT would be recommendable for the effect of this intervention over time. Further, while we know that patients and relatives valued the consultations, we do not know why patients and patients’ relatives with spiritual issues who were offered but did not use the consultations, did not participate. Finally, this study does not provide any insights into the relatively high use of consultations by relatives instead of patients. We recommend these issues for future research.

## Conclusion

If sufficient effort and time is given to implementation, the listening consultation service can be a good method for PaTz-groups to cooperate with spiritual caregivers, to receive training in spiritual care skills and to refer patients and relatives with spiritual needs to spiritual caregivers. Listening consultation services could also serve as a good method for integrating spiritual care in primary palliative care other contexts, such as multidisciplinary meetings between healthcare professionals in other countries or contexts.

## Supplementary information

**Additional file 1.** Topic lists of semi-structured individual interviews with spiritual caregivers

**Additional file 2.** Practical recommendations for interventions concerning integration of spiritual care in palliative care

## Data Availability

The datasets generated and/or analysed during the current pilot study are not publicly available due to the small scale of this pilot study and the easily traceable nature of the data, but are available from the corresponding author on reasonable request.

## Abbreviations

PaTz-group: Palliative home care groups
WHO: World Health Organization
EAPC: European Association for Palliative Care
GP: General practitioner
PaTz: Palliatieve ThuisZorg (Palliative Care at Home)
COREQ: Consolidated criteria guidelines for reporting qualitative studies
VGVZ: Dutch Association of Spiritual Caregivers
SKVG: Quality Register of Spiritual Caregivers
POH-GGZ: Nurse specialist in mental health
RCT: Randomized Controlled Trial
