# Supplementary material for: Strengthening the spiritual domain in palliative care through a listening consultation service by spiritual caregivers in Dutch PaTz-groups: an evaluation study
Source: BMC Palliat Care. 2020 Jun 29;19:92. doi: 10.1186/s12904-020-00595-0 (PMC7325007; doi:10.1186/s12904-020-00595-0)
Supplement: Supplementary file 1 — Additional file 1. Topic lists of semi-structured individual interviews with spiritual caregivers [file 12904_2020_595_MOESM1_ESM.pdf]

Topic lists used in group and individual interviews.

Group interviews: GPs, district nurses and consultant palliative care

- Short introduction of the subject
- Providing listening consultation services
  - Attitude towards utility
  - Care request of patient
- Referral
  - Reasons for referral and non-referral
  - Practical considerations
- Experiences with consultations
  - Patients' experiences
  - PaTz-group members' experiences
  - Experienced added value
- Experiences with participation of spiritual caregivers in PaTz-groups
  - Contribution to group discussions
  - Experiences regarding collaboration with spiritual caregivers
  - Experiences with (own) attention for spiritual domain
  - Effect on knowledge on / experiences with spiritual care for patients or relatives at the end of life
  - Experienced added value

Individual interviews: Spiritual caregivers

- Short introduction of the subject
- Providing listening consultation services
  - Attitude towards utility
  - Referrals in practice
  - Discussed topics
- Experiences with consultations
  - Patients' experiences
  - Spiritual caregivers' experiences
  - Added value
- Experiences with participation in PaTz-groups
  - Contribution to patient discussions
  - Collaboration with other healthcare providers
  - Attention for spiritual domain
  - Added value

Individual interviews: Patients and relatives who used consultation services

- Short introduction of the subject
- Reasons for using listening consultation services
  - Care request
  - Referral
- The consultation(s)
  - Discussed topics
  - Attitude of spiritual caregiver
  - Added value
  - Effects in daily life or feelings
  - Attention for spiritual care in palliative care
