# Supplementary material for: Strengthening the spiritual domain in palliative care through a listening consultation service by spiritual caregivers in Dutch PaTz-groups: an evaluation study
Source: BMC Palliat Care. 2020 Jun 29;19:92. doi: 10.1186/s12904-020-00595-0 (PMC7325007; doi:10.1186/s12904-020-00595-0)
Supplement: Supplementary file 2 — Additional file 2. Practical recommendations for interventions concerning integration of spiritual care in palliative care [file 12904_2020_595_MOESM2_ESM.pdf]

1. Agree who takes initiative for the (listening consultation) services

- Appoint a ‘champion’ who knows and understands both fields of healthcare and spiritual care
- Select a ‘champion’ who is willing to invest time in the initial phase of the project
- Discuss an appropriate title for the initiative with all involved stakeholders

2. Find an motivated, enthusiastic group of healthcare professionals (e.g. PaTz-group or multidisciplinary group)

- Select a group or chairmen who is motivated for integrating spiritual care into palliative care
- Seek for a group with frequent meetings

3. Ensure a good match between the spiritual caregiver and the group

- Determine (e.g. with the chairmen) which “color” or denomination spiritual caregiver is needed

4. Prepare the start well

- Make customized brochures and hand them out to each group member
- Agree on a clear route of referral of healthcare professionals to the spiritual caregiver
- Agree on a financial route of reimbursements: who pays the spiritual caregiver?
    - Think of: rate for consultations, rate for training, rate for participation in group meetings
    - Travel time compensation / travel costs reimbursement
    - Reimbursement for time spend to calling patients for scheduling a consultation

Agree on a start date for the consultations, group meeting and training

- Define the target group (of listening consultation services)
- Think of: seriously ill patients with chronic disorders, patients’ relatives, patients with a wish for euthanasia
- Agree on form of consultations: walk-in hour or home visits and on route of referral: by patients or by professionals?
- Agree on (not) sharing personal data
- Agree on which terms you use and use them unambiguously, e.g. “spiritual caregiver”, “chaplaincy”

5. Invest in collaboration and get to know each other

- Arrange an appointment between the chairman / group and spiritual caregiver
- Make an inventory of whether the spiritual caregiver is available on data of group meetings
- Provide each group member with contact details of the spiritual caregiver (business card / brochure)
- Do an inventory of involved healthcare professionals’ vision on spiritual care
- Define role of spiritual caregiver and manage expectations: consultations, group attendance, intervision?
- Inform group members on spiritual care
- Ask for specific needs for training

6. Invest in a good start and further collaboration

- Make clear for all involved professionals when consultations can be used
- Use flyers to communicate
- Ensure findability of spiritual caregivers: can he / she be found by everyone?
- Use posters and flyers to communicate about the services
- Print and distribute flyers regularly
- Let group members actively offer listening consultation services (by a coordinator)
- Send a newsletter regularly or put a news message on a website (by a coordinator)

7. Continue to invest: training and customization

- Offer training and adjust duration of training to the needs of group members
- Repeat training if desired
- Plan group meetings in close collaboration with the spiritual caregiver as a group member
- Send the agenda of the group meeting to the spiritual caregiver beforehand
- Make sure the spiritual caregiver attends meetings regularly

8. Provide feedback on a regular basis

- Agree whether the referrer(s) wants feedback and how you eventually arrange giving feedback (e.g. phone, e-mail)
- Agree that spiritual caregivers ask the patients’ permission for providing feedback to referrer

9. Sustainability and finance

- Embed the listening consultation services in a local network, e.g. Palliative Care Network
- Discuss whether there is a maximum number of consultations
- Match how consultations / services are paid, e.g. on a claim basis
- Coordinate how a spiritual caregivers keep being involved (e.g. by the Center for Meaning Questions)
